# Factors influencing the retention of secondary midwives at health centres in rural areas in Cambodia: the role of gender—a qualitative study

**DOI:** 10.1186/s12913-021-07239-w

**Published:** 2021-11-19

**Authors:** Kimiko Abe, Bandeth Ros, Kimly Chea, Rathavy Tung, Suzanne Fustukian

**Affiliations:** 1 Liberal Studies, Jissen Women’s University, Tokyo, Japan; 2 Freelance researcher, Phnom Penh, Cambodia; 3 University of Medical Science, Phnom Penh, Cambodia; 4 National Maternal and Child Health Centre, Phnom Penh, Cambodia; 5 Institute for Global Health and Development, Queen Margaret University, Edinburgh, UK

**Keywords:** Midwives, Retention, Rural areas, Gender, Low- and middle-income countries, Post-conflict, Cambodia

## Abstract

**Background:**

Retention of skilled midwives is crucial to reducing maternal mortality in rural areas; hence, Cambodia has been trying to retain at least one secondary midwife who can provide basic emergency obstetric care at every health centre even in rural areas. The factors influencing the retention of midwives, but not solely secondary midwives, have been identified; however, the security issues that affected female health workers during the conflict and the post-conflict years and gender issues have been unexplored. This study explores these and other potential factors influencing secondary midwife retention and their significance.

**Methods:**

Sequential two-stage qualitative interviews explored influential factors and their significance. The first stage comprised semi-structured interviews with 19 key informants concerned with secondary midwife retention and in-depth interviews with eight women who had deliveries at rural health centres. Based on these interview results, in-depth interviews with six secondary midwives who were deployed to a rural health centre were conducted in the second stage. These midwives ranked the factors using a participatory rural appraisal tool. These interviews were coded with the framework approach.

**Results:**

Living with one’s parents or husband, accommodation and security issues were identified as more significant influential factors for secondary midwife retention than current salary and the physical condition of the health centre. Gender norms were entrenched in these highly influential factors. The deployed secondary midwives who were living apart from one’s parents or spouse requested transfer (end of retention) to health centres closer to home, as other midwives had done. They feared gender-based violence, although violence against them and the women around them was not reported. The health workers surrounding the midwives endorsed the gender norms and the midwives’ responses. The ranking of factors showed similarities to the interview results.

**Conclusions:**

This study suggests that gender norms increased the significance of issues with deployments to rural areas and security issues as negative factors on female health workforce retention in rural areas in Cambodia. This finding implies that further incorporating gendered perspectives into research and developing and implementing gender-responsive policies are necessary to retain the female health workforce, thereby achieving SDGs 3 and 5.

## Background

In rural areas, the shortage of human resources for health (HRH) has presented significant obstacles to universal health coverage, particularly in post-conflict and fragile countries [1–3]. In many post-conflict countries, active conflicts have ceased for a variety of reasons, and these countries often undergo transformation to achieve post-conflict political settlement [4]. In fragile post-conflict countries, the government frequently lacks the capacity or will to perform basic functions such as providing basic services or bringing in access to services or equal job opportunities [5]. Recruiting and retaining HRH in such settings have been attempted by various policy measures [6, 7]. However, gender issues, among other factors, have been increasingly identified as presenting significant challenges to the retention of female HRH globally, especially in rural areas in many low- and middle-income countries (LMICs) [8–12]. The identified gender issues faced by female HRH include gender-based violence [9, 10], gender-based rejection [12] and the responsibilities of caring for family members [9].

Following nearly three decades of war between 1970 and 1998 and particularly since the general election in 1993, the Ministry of Health (MoH) of Cambodia, a fragile LMIC, fostered development, increased the amount of HRH and succeeded in reducing the country’s high maternal mortality ratio from 484 to 100,000 live births in 2000 to 161 per 100,000 live births in 2015, achieving Millennium Development Goal 5.A [13]. Included among the various factors that contributed to this reduction [14] were those related to rebuilding the country’s decimated HRH and, in particular, the enhanced production of midwives, both primary and secondary, after 2005, as highlighted by Fujita et al. [15]. This was accompanied by improved remuneration and more widespread deployment of these cadres, with at least one primary midwife deployed to every health centre (HC), even in rural areas [15].

The Cambodian MoH, aiming at further reductions in the maternal mortality ratios, launched a policy to deploy at least one of the more qualified secondary midwives (SMWs) to every HC in 2010, since SMWs can provide emergency obstetric and newborn care [16]. This policy was modified to deploy two SMWs to designated HCs that performed specific emergency obstetric and newborn care [17]. However, despite the MoH’s efforts, some HCs, particularly those in rural (and often poor) provinces, have yet to receive an SMW [18]. Qualification as a SMW requires an associate degree (three years) in midwifery and this degree was introduced in 2008 [15]. Qualification as a primary midwife required one year of midwifery education, but the course existed only until 2015 [19]. These two grades of midwives, primary and secondary midwives, were introduced by the MoH in the 1980s, as the MoH accelerated the production of HRH after the Khmer Rouge genocide. The MoH then enacted changes in the prerequisite education, the length of midwifery education and required diplomas and degrees. Therefore, it should be noted that SMWs include those who were educated according to the old curriculum before the introduction of the associate degree. Their midwifery education was shorter than three years, including, for example, one year of midwifery education after three years of nursing education [15].

After completing their education, when the midwifery graduates take the MoH examination to obtain a civil servant post, they simultaneously apply to their respective provincial health departments, not a specific HC. After the examination, the health department assigns SMWs to vacant or new posts at HCs or other MoH-affiliated facilities. However, in rural areas, houses for rent are rare [20], and the MoH’s HRH policy does not include a housing allowance [21].

Previous studies and the grey literature have identified the potential factors that influence the retention of SMWs; however, they have not specifically studied SMWs, focusing on midwives more broadly. The identified factors include midwives’ salaries, which are lower than those paid to SMWs by non-governmental organisations (NGOs) [22]; the physical conditions of health facilities, including shortages of medical equipment and medicines [23]; and technical guidance and advice from superiors [18, 22].

The MoH and local health officials have stated their concerns about security issues, namely, violence against women deployed as SMWs (personal contacts, anonymous, on November 12, 2008, September 6, 2011, and August 18, 2012). Such concerns are in line with previous research on cases of violence against female HRH in LMICs [8]. Importantly, in Cambodia, security problems for female HRH and the stigmatisation of victims occurred in the conflict and post-conflict years [24], and violence against women by men in Cambodia has been prevalent, but such cases have been underreported [25]. Moreover, gender norms tend to restrict aspects of women’s lives; living with one’s parents before marriage, preserving one’s virginity until marriage, and living with a spouse after marriage are highly important to Cambodian women [26]. Gender norms have kept the status of women much lower than that of men in Cambodia, as evidenced by the global gender gap index [27].

To date, no study has investigated how the aforementioned factors and other factors affect the desire of SMWs to remain in their posts at rural HCs. Therefore, the present study aims to explore the factors that influence the retention of SMWs, especially at rural HCs in Cambodia, and compare the significance of these factors to seek effective retention policies.

## Methods

The qualitative research in this study drew on Lincoln and Guba (1985), who described the trustworthiness of qualitative findings as constituted by four aspects: credibility, transferability, dependability and confirmability. Strategies to ensure trustworthiness [28] were implemented as described in this section.

### Setting

This study was conducted in Phnom Penh and the rural areas of Kampong Cham Province in February and August 2017. The province is known for its agriculture and vast areas of rubber and other plantations that receive internal migrant workers. The presence of unpaved roads and rivers are causing transportation difficulties within the area. HCs in rural areas, particularly those that are located away from national roads or provincial/district towns or main markets, are affected by transportation difficulties and poverty.

### Study design

This study consisted of sequential two-stage qualitative interviews [29] and the use of a participatory ranking tool [30] to identify highly influential factors for SMWs’ retention at rural HCs.

### Interviews

 Interviews were conducted in either English or/and Khmer using the interview guides tailored to each type of interviewees and ranged from 1 to 1.5 h in length. The interviews included key informant, semi-structured and in-depth interviews [28]. Interviewees were purposively selected. Pretests were conducted with individuals who shared attributes with this study participants but who were not actual subjects [31]. With the interviewees’ consent, most interviews were recorded. Detailed notes about the interviews were taken by interviewers during all interviews [32]. A debriefing by interviewers, including interpreters, was conducted after each day of data collection [33].

### First stage of the research

Semi-structured interviews were conducted with key informants in the first stage of the research. The key informants were managers of strategically selected stakeholder organisations at the national and regional levels involved in the deployment and/or retention of SMWs as well as those involved in the education and training of SMWs. Key informants at the national level included managers who were concerned with SMWs’ deployment and retention: the managers at the MoH, the National Centre for Maternal and Child Health, bilateral and multilateral development partners and international and national NGOs. These organisations were identified as being engaged in the function of deployment or retention in the ‘house model’ for HRH system development, specifically formulated for the Cambodian health sector [15]. In this model, education and training belong to the function of HRH ‘production’ and managers who were engaged in the education and training of SMWs were considered to have relevant experience concerning midwifery students’ or midwives’ preferences regarding deployment and retention. Therefore, we included a manager at the Regional Training Centre affiliated with the MoH as a key informant at the regional level.

We purposively selected the key informants at the community level in rural areas. These key informants were those who worked closely with SMWs and were presumably aware of community attitudes towards the SMWs; they included managers in charge of reproductive, maternal, newborn and child health of the operational (health) district to which the SMWs’ HCs belonged and the respective HC chiefs and female volunteers collaborating with the HC.

They were selected through the following steps. First, the HCs were chosen based on their deployment of SMWs, where both SMWs and HCs met our defined sampling criteria. Specifically, the HCs were required to have at least one SMW (with a three-year midwifery associate degree) who was aged 30 years or younger and who had been deployed in the seven years preceding 2017 (because the MoH began to apply the policy for deploying SMWs to HCs in 2010) [16]; in addition, the HCs had to be located away from national roads, provincial/district towns and main markets. After HCs that met these criteria were identified, the operational districts that had jurisdiction over these HCs and these districts’ mangers in charge of reproductive, maternal, newborn and child health, were selected, as were the HC chiefs and female health volunteers who were selected as the key informants.

To find these community-level interviewees, we consulted the Health Department of Kampong Cham Province. The health department introduced us to the offices of operational districts within the province that had jurisdiction over the HCs to which the SMWs were deployed. These HCs fulfilled our selection criteria in terms of both their locations and the attributes of their deployed SMWs, and thus the operational district offices introduced us to their own managers in charge of reproductive, maternal, newborn and child health and the HC chiefs, who in turn introduced us to female volunteers.

During the semi-structured interviews, these key informants as well as the other key informants at the national and regional levels were asked about the following factors that influenced the retention of SMWs: physical conditions of the HCs, technical guidance and advice from superiors, security issues and gender [8–12, 18, 22–24].

In addition to these key informants, we selected women who had deliveries at the identified HC over the past 12 months for the in-depth interviews, since these women had presumably received health services from and had interactions with the SMWs and were also aware of community attitudes towards the SMWs. The HC chiefs we interviewed also introduced us to these women, and in the interviews, we asked them about their experiences related to pregnancy, delivery and the retention of the SMWs they interacted with.

After each interview was conducted, it was transcribed separately in the two languages, and the two versions were compared to include information spoken in Khmer that was missing in the English version. Drawing on the framework approach [34], one of our researchers repeatedly read and coded the revised English versions (using NVivo 11) according to the following themes: salary, the physical condition of the HCs, technical guidance and advice from superiors, security issues, and gender [8–12, 18, 22–24]. Then, these codes were categorised. Moreover, open coding was applied to identify additional themes and sub-themes, and these codes were also categorised.

Next, a series of reviews was performed during which the categories for the codes, the themes and the sub-themes were revised, and each revised item in these groups was compared to the others both within a group and among the three groups in consultation with another researcher until the researchers agreed upon the categories, themes and sub-themes. Through this process, important influential factors were derived. In addition to these repetitive steps [34] taken by the two researchers, all authors with different backgrounds discussed the results of the data analysis, including the results written in the various drafts of this paper, until the authors agreed upon the results [35].

### Second stage of the research

The second stage of the research involved in-depth interviews with SMWs holding a three-year-associate degree who met the aforementioned criteria: aged 30 years or younger; deployed to one of the HCs located away from national roads, provincial/district towns and main markets; and deployed in the seven years preceding 2017 (because the MoH began to apply the policy for deploying SMWs to HCs in 2010) [16].

To identify the SMWs who met these selection criteria, we consulted the Health Department of Kampong Cham Province, as we did in the first stage of research, and which introduced us to the suitable SMWs. Furthermore, we applied the snowball sampling method to identify more SMW interviewees who met the selection criteria.

These interviews explored the SMWs’ experiences being deployed to and working at rural HCs as well as the significance of both the factors we asked the key informants about and any other factors that were influential and important to their retention, reflecting the results of the first-stage interviews. The interviews were analysed using the same process as the first-stage interviews, and the results were synthesised with the first-stage results.

**Fig. 1:**
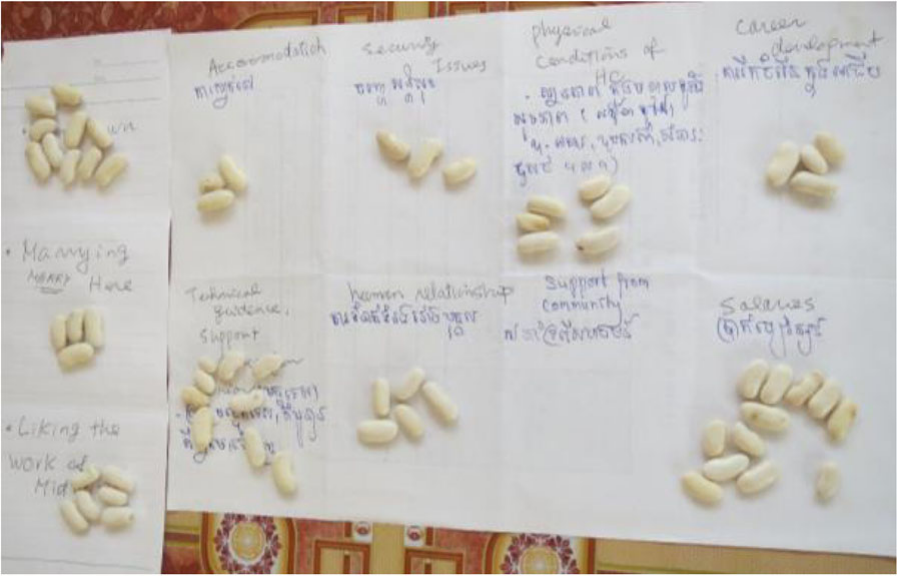
A sheet with 11 grids (factors) for ranking the factors’ significance.

After each interview, the SMWs were asked to rank the significance of the factors that were discussed in their interviews and those that they perceived or mentioned as influential and important in the interviews. For this purpose, *each* of these factors was written in a grid on a sheet, and the SMW placed different quantities of beans on the different factors to show the corresponding significance of each factor (Fig. 1).

## Results

The first-stage interviews were conducted with 19 key informants (Table 1) and eight women (Mother 1–8, two from each of the four HCs). The key informants at the national and regional levels were managers of these organisations (Table 1).

**Table 1:** Organisations and attributes of the interviewed key informants

Level	Function	
	**Deployment**	**Retention**
National	MoH (*N* = 1) Development Partner (*N* = 1)	National Centre for Maternal and Child Health (*N* = 1) Development Partners (*N*=3) NGOs (*N* = 4)
	**Production**	
Regional	Regional Training Centre (*N* = 1)	–––––
Community & HC	Manager for reproductive, maternal, newborn and child health of operational districts (*N* = 1) HC chief (*N* = 4) Female village volunteers (*N* = 3)	

We interviewed eight SMWs. The earliest year that one had begun work at her current HC was 2013, and the most recent year was 2016. Three SMWs were single, and only one was from outside of the focal province. We carefully excluded midwives who had completed only one year of midwifery education after three years of nursing education to obtain an associate degree in nursing and midwifery courses, who were frequently also called secondary midwives.

### Deploying SMWs to unpopular HCs

The responses to the interviews contained additional essential information regarding the deployment of SMWs to rural HCs. For example, some provincial health departments required SMWs to draw lots to decide who would work at unpopular HCs rather than directly assign them to such locations. Based on the lottery results or assignments, some SMWs were deployed to HCs that were located far from their hometowns or in entirely unfamiliar locations. Importantly, SMWs were contracted by a provincial health department to work at their first post for at least five years. After a five-year posting at a single site, the legislation allows civil servants to transfer to another location.

### Living with parents or spouses and accommodation

Both the key informants at the community level and the SMWs interviewed agreed that having to live separately from their parents or spouses due to their deployment influenced SMWs to transfer to another HC (i.e., the end of retention) to be able to live with their parents or spouses, even before the end of the five-year contract. These SMWs made a request to the operational district office, not the provincial health department, for their transfer; in some cases, if the first transfer request was not fulfilled, the SMWs would make a second request (SMW 3). These interviewees and SMWs also mentioned similar situations at other HCs. Three out of the six SMWs interviewed either were granted their transfer requests or made additional requests (SMW 3, 4, 6). In this regard, one key informant, a government manager stated the following:

*“As you know, midwives are female. If those midwives are not yet married, their parents often do not encourage them to stay in such a place.”* (Government Manager 1).

In other cases, since some SMWs did not move to the community where the HC they were deployed to was located to avoid living apart from their parents, the other midwives in the same HC volunteered for the on-call night duties of these SMWs.

A key informant, a manager at an international NGO who conducted reproductive health projects mentioned complaints she received from Cambodian parents regarding their daughters’ overnight business trips within the country (NGO Manager 3).

### Accommodations of the SMWs

Interestingly, the key informants at the community level agreed that SMWs deployed outside their home communities should live with families that had either no male family members or only prepubescent male family members. In this regard, the SMWs who were living separately from their families because of their deployment stated that they were either living or had lived with elderly widowed women who had grandchildren who were either girls or prepubescent boys. Notably, some HC chiefs helped the SMWs find such accommodations.

Three out of the four HC chiefs interviewed accepted that the SMWs’ departure from their HCs was inevitable despite the support offered to the SMWs because the SMWs did not live with their families in their hometowns. The SMWs also mentioned that they appreciated the support from their respective HC chiefs and others in the community, but their eagerness to transfer surpassed such appreciation.

### Security issues

Concerns about security issues with respect to SMWs’ deployment in rural areas were also shared by the two government managers interviewed (Government Manager 1, 4). However, no security incidents targeting women, including SMWs, were reported in any of the interviews, except for one, which may have been a special case. This finding indicates that security issues may not have been a significant factor in SMWs’ rural deployment. Regarding the special case, one female volunteer stated the following:

*“One woman (who was living by herself in a village) got pregnant without a husband…The woman has some mental problems. …a small proportion of the community pitied her. But the majority blamed her.”* (Female Volunteer 2).

This volunteer also stated that many people in the community believed that the woman had made herself vulnerable to sexual harassment or assault because she lived by herself (Female Volunteer 2). This story exemplifies the gender norms in Cambodia that often exacerbate women’s difficulties, including victim blaming. Despite the absence of security incidents, the key informants at the community-level, the women interviewed who had deliveries at the HCs, and the SMWs persistently expressed anxiety about security issues for the midwives, as indicated by the following:

*“There is always a concern over security problems, although we have never heard of any security problems in this community*.*”* (Mother 3).

### Economic remuneration

Salaries, including delivery incentives, were considered adequate by the SMWs. However, this finding contradicted the view of several key informants who stated that SMWs’ salaries were a highly influential factor affecting their retention. In some cases, the SMWs compared their salaries and workloads at private clinics with those at the HCs. These SMWs had previously temporarily worked for private clinics during the time between graduating from midwifery school and obtaining a government post. They agreed that their previous salaries had been generally higher but that their workloads had been much heavier than at the HCs.

Some key informants who belonged to either the government, development partners, or NGOs mentioned that the SMWs working in rural areas preferred a more relaxed work environment with better job security, which they currently enjoyed, even though their salaries were lower than those that they would have been paid by NGOs. They added that these SMWs rarely transferred to NGOs, where jobs are often demanding.

### Additional factors

Some of the midwives occasionally felt dissatisfied with the physical conditions of the HCs (for example, overcrowding in the maternity ward when three deliveries occurred simultaneously or flooding in the front yard of an HC after a heavy rain), which may have influenced their retention. However, the SMWs did not complain about a shortage of technical learning and appreciated having informal and formal opportunities to learn and develop their skills from their superiors. All the SMWs also stated that they considered good human relationships within the HCs to be an important factor that influenced their retention. Moreover, the SMWs rarely described any negative actions taken by their superiors or colleagues.

Finally, all the SMWs and the interviewed women agreed that the women followed the SMWs’ advice, even when the advice differed from or contradicted the Cambodian customs and practices related to pregnancy and delivery or prohibited them from following these customs and practices. The interviewed women also expressed that their own mothers and the people in the community did not reject but accepted the SMWs’ advice.

### Ranking of influential factors by the SMWs

**Fig. 2:**
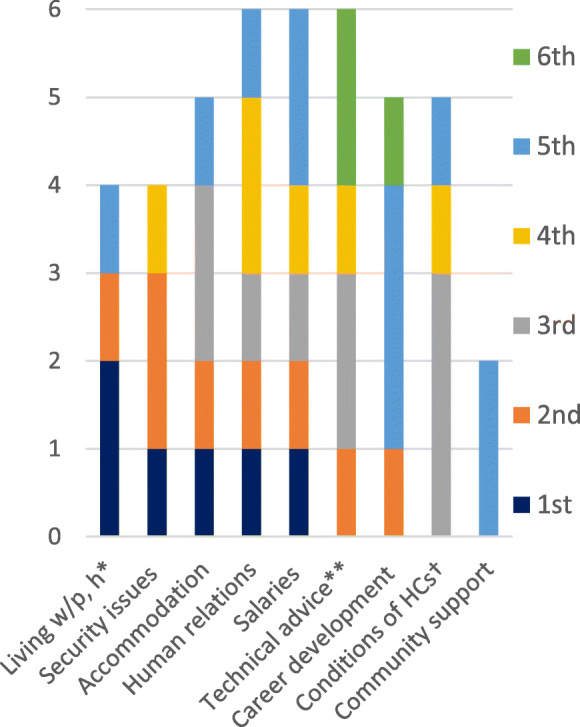
Frequency of rankings (1st through 6th) of factors. Note: Living w/p, h * = Living with parents or husband; Technical advice** = Technical advice and guidance; Conditions of HCs† = Physical conditions of the HCs.

The maximum number of influential factors that the SMWs ranked was 13 factors. However, not all SMWs included all the factors in their rankings. The bars in Fig. 2 are the nine important influential factors, that were ranked by two and more SMWs. The area within each bar in different colours represents the frequency of the ranking (limited to 1st through 6th ) assigned by the SMWs. ‘Living with parents or husband’ and ‘security issues’ (the first two bars on the left side) were ranked as the most important factors. These results are in line with the interviews with the SMWs and the key informants. However, one source of uncertainty in this ranking activity was the difference in the total number of factors ranked by each SMW. Thus, the results provide only corroborative evidence.

## Discussion

The retention of SMWs was found to be largely influenced by gender norms rather than the previously identified factors. Living separately from one’s parents or spouse due to deployment was the most important factor influencing SMW retention, even more important than the SMWs’ salaries or the physical conditions of the HC, which had been improved by Cambodian HRH policies [14]. The lower importance given to salaries was in contrast to the perception that SMWs tend to transfer to NGOs to obtain higher remuneration than that offered by the government [22].

Separation from the family influenced the SMWs to make transfer requests, i.e., ending the retention of the SMWs at their deployed HCs. Their requests could be largely attributed to gender norms. Living with one’s parents before marriage, preserving one’s virginity until marriage, and living with one’s spouse after marriage are highly important to Cambodian women [26]. Such norms appeared to prevail among the HC staff members and those around them, including the SMWs’ parents and spouses.

These findings support previous findings in both LMICs and high-income countries that showed that gender norms hindered the retention of female HRH [11, 9].

Our study found that searching for appropriate accommodations that align with gender norms posed an extra burden on the SMWs and those supporting them. This burden would affect the retention of SMWs, and no official policies for housing allowances (in kind or in cash) have been implemented [21].

These findings also revealed a structural issue that was consistent with a previous finding on the lower retention of female HRH than male HRH in countries in the European Union, which suggested that the lower retention is brought about by societal expectations of female HRH rooted in gender norms and gender-responsive institutional policies that are lacking or inadequate [9].

Establishing a dormitory where SMWs could live together under the supervision of a dormitory manager and his or her family or a gatekeeper who also works as a guard, known as *nak yam*, may be an alternative and reduce the fear of security issues identified in this study; this will be discussed below.

The mismatches between the SMWs’ hopes and the locations of the HCs to which they were deployed may also be due to the limited number of newly graduated SMWs from remote rural villages, where a low proportion of women enrol in secondary education (a prerequisite for midwifery education) [35] due to gender norms [26].

Security issues were also an important factor that influenced the retention [9, 10] of SMWs, even though security incidents involving SMWs or other women in the community were rare. However, the SMWs, those around them, and some officials feared that violence against women could still occur for SMWs deployed to rural HCs. This contradictory and disproportionate significance of security issues could be largely due to the gender norms in Cambodia [26] for female HRH that were prevalent in the 1990s, the post-conflict era, and that hindered their ability to work night shifts or be deployed to a facility far from home [24]. Such gender norms tend to persist, particularly in rural areas. The story told by a female volunteer about the woman who lived alone exemplifies the types of gender norms that often exacerbate women’s difficulties, including victim blaming. This story suggests that gender norms still restrain women, including SMWs, from moving and living alone [26] in rural areas; in addition, it corroborates our finding that SMWs have a disproportionate fear of security issues and that security issues have higher significance in their retention, both of which would be attributed to gender norms. Importantly, although the reporting of incidents to police has been low amidst the high prevalence of violence in Cambodia [25], we cannot completely support the SMWs’ and other interviewees’ responses that mentioned almost no occurrences of violence against women in their community.

Interestingly, the present study found limited dissatisfaction among SMWs regarding their economic remuneration and the physical conditions of HCs. Their feelings could be due to salary increases and the introduction of delivery incentives by the Cambodian government in the early 2000s and previous improvements in the physical conditions of the HCs [14]. In some cases, the SMWs’ satisfaction with their current remuneration was underpinned by their previous experiences working for private clinics.

Finally, the relatively high significance of human relationships may have been reflexive and innocuous and reflected a cliché among SMWs that having good human relations is important in the workplace. Furthermore, since the rejection of SMWs’ health advice by pregnant women, their mothers and senior people in the community was denied in the interviews, it could be argued that the rejection of SMWs’ advice by their patients and people in the community had a limited effect on their retention, contrasting with the findings of other studies [12].

A limitation of this study is the lack of perspective from provincial health departments on influential factors in SMW retention, even though provincial health departments are responsible for assigning SMWs to HCs within the province. Further research should include their perspective to deepen the understanding of the significance of influential factors. Another limitation of this study is that the number of SMWs who participated in the ranking of the influential factors was small; consequently, the ranking results remained corroborative, with a decreased effect of triangulation by this quantitative method. Future studies should increase the number of participants so that their numbers are sufficient to quantitatively investigate the ranking of significant factors.

## Conclusions

This study found that the retention of SMWs, especially at rural HCs, in Cambodia was largely influenced by gender norms rather than by salaries or physical conditions of the HC, which were previously identified as important influential factors. Gender norms that were embedded in factors such as living away from home, accommodation and security issues prompted SMWs to officially request a transfer from their current HC (ending of retention) to HCs closer to home. The MoH could respond to the factors affected by gender norms by providing a housing allowance to SMWs deployed in rural areas. This study also suggests that reviewing current HRH issues from a gendered perspective and implementing gender-responsive HRH policies could further reduce the difficulties faced by female HRH deployed in rural areas, which ultimately could result in the attainment of SDGs 3 and 5.

## Data Availability

The datasets generated and/or analysed during the current study are not publicly available to protect the anonymity and privacy of the interviewees but are available from the corresponding author on reasonable request.

## Abbreviations

HC: health centre
HRH: human resources for health
LMICs: low- and middle-income countries
MoH: Ministry of Health
NGOs: non-governmental organisations
SMW: secondary midwife
